# Fundamental Study on the Fabrication of Inverted Planar Perovskite Solar Cells Using Two-Step Sequential Substrate Vibration-Assisted Spray Coating (2S-SVASC)

**DOI:** 10.1186/s11671-016-1259-2

**Published:** 2016-02-05

**Authors:** Fatemeh Zabihi, Mohammad-Reza Ahmadian-Yazdi, Morteza Eslamian

**Affiliations:** University of Michigan-Shanghai Jiao Tong University Joint Institute, Shanghai, 200240 China

**Keywords:** Planar perovskite solar cells, Thin films, Spray coating, Ultrasonic substrate vibration, Substrate vibration-assisted spray coating (SVASC)

## Abstract

In this paper, a scalable and fast process is developed and employed for the fabrication of the perovskite light harvesting layer in inverted planar heterojunction solar cell (FTO/PEDOT:PSS/CH_3_NH_3_PbI_3−*x*_Cl_*x*_/PCBM/Al). Perovskite precursor solutions are sprayed onto an ultrasonically vibrating substrate in two sequential steps via a process herein termed as the two-step sequential substrate vibration-assisted spray coating (2S-SVASC). The gentle imposed ultrasonic vibration on the substrate promotes droplet spreading and coalescence, surface wetting, evaporation, mixing of reagents, and uniform growth of perovskite nanocrystals. The role of the substrate temperature, substrate vibration intensity, and the time interval between the two sequential sprays are studied on the roughness, coverage, and crystalline structure of perovskite thin films. We demonstrate that a combination of a long time interval between spraying of precursor solutions (15 min), a high substrate temperature (120 °C), and a mild substrate vibration power (5 W) results in a favorable morphology and surface quality. The characteristics and performance of prepared perovskite thin films made via the 2S-SVASC technique are compared with those of the co-sprayed perovskite thin films. The maximum power conversion efficiency of 5.08 % on a 0.3-cm^2^ active area is obtained for the device made via the scalable 2S-SVASC technique.

## Background

Lead halide methylammonium perovskite is an emerging and promising light-harvesting material developed for the fabrication of emerging photovoltaic solar cells (SCs). The power conversion efficiency (PCE) of perovskite solar cells made using lab-scale deposition methods has increased rapidly in a few years exceeding an impressive PCE of 20 % [1–6]. This is due to the excellent optoelectronic properties of perovskites, such as appropriate direct bandgap, high absorption coefficient (low required film thickness), the ability for photon absorption in a wide range of the spectrum, ambi-polarity, efficient carrier transportation, and delivering high open circuit voltage [5]. An outstanding capability of perovskites is the dual function of light harvesting and hole-electron conducting with a low exciton binding energy. It was systematically proved that the Fermi level of perovskite lies near the conduction band edge, indicating that the perovskite is inherently a weak n-type semiconductor which may show p-type behavior [7, 8]. Because of the aforementioned features of perovskites, the perovskite solar cell can outperform the polymeric and dye-synthesized solar cells [1–9]. However, issues such as the lifetime, stability, and compatibility with the large-scale and roll-to-roll fabrication processes are yet to be addressed. It is also noted that even in the lab-scale, it is hard to control the reaction between the perovskite reagents, and crystallization mechanism and the morphological characteristics of the perovskite layer [10]. Below is a brief review of the breakthroughs in the field of perovskite solar cells.

Kojima et al. were the first who reported the application of methylammonium lead iodide perovskite (CH_3_NH_3_PbI) as a sensitizer for mesoporous TiO_2_ film in an electron/hole-selective free solar cell [11]. The ensuing device demonstrated PCE of 3.8 % but suffered from low stability. By embedding a hole-selective semiconductor in the cell, the PCE was significantly raised with much better stability [1, 10, 12–14]. Application of the mixed-halide perovskites (CH_3_NH_3_PbI_3−*x*_Cl_*x*_), infiltrated into mesoporous TiO_2_ and Al_2_O_3_ layers, resulted in further improvement in PCE and stability of perovskite SCs [15–19]. The mixed-halide perovskites exhibit a rather long diffusion length, which is tunable in different cell architectures [15–18]. The Cl^−^ anions in CH_3_NH_3_PbI_3−*x*_Cl_*x*_ can boost the mobility of excitons and the charge carrier transport [16–19]. In addition to the traditional mesoporous architectures, perovskite solar cells with planar heterojunctions have been proposed to reduce the production costs and difficulties associated with the fabrication of mesoporous layers. In this configuration, the active layer is deposited on a flat substrate (electrode or hole-electron-selective layers) [3–6, 20–23]. In the planar structures, the mixed-halide perovskites are preferred because the charge diffusion length of the mixed-halide perovskites can be larger than the depth of the light absorption, which is the film thickness in planar structures [18–22]. Deposition of perovskite thin film on a flat substrate guarantees a higher internal quantum efficiency; however, the lower performance associated with this type of devices compared to mesoporous devices is due to the structural imperfections, such as pinholes, non-homogenous crystal growth, and low coverage associated with planar thin films. The low coverage and non-uniform crystalline network may result in low-resistance shunting paths, reducing the maximum attainable open circuit voltage and fill factor [5, 20–25]. The poor surface coverage causes the incident photons to pass straight through the uncovered areas, decreasing the available photocurrent; also, the excessive shunt paths due to direct contact between the hole-transporting material (HTM) and electron transporting material (ETM) lead to the formation of parallel diodes in the solar cell equivalent circuit [5]. Due to the above-mentioned problems associated with planar structures, the application of mesostructured perovskite devices would still yield a higher PCE; however, such devices are not easy to commercialize due to the requirement for thermal processing at high temperatures [24]. Thus, in this work, and in an attempt to pave the pathway for successful commercialization of perovskite solar cells, the inverted planar architecture is adopted combined with spray-coating fabrication method, which is a scalable technique.

Many efforts, such as solvent engineering, chemical tuning, and controlling over crystallization and chemical conversion are being undertaken to improve the performance of inverted and conventional planar perovskite solar cells and to suppress the performance of mesoporous architectures. For instance, Jeng and co-workers developed a donor-acceptor planar SC by growing a thin layer of C_60_ on CH_3_NH_3_PbI_3_ [25]. The hybrid cell delivered the highest PCE of 3.9 %. Barrows et al. [21] reported the use of ultrasonic spray coating of CH_3_NH_3_PbI_3−*x*_Cl_*x*_ in a planar structure with organic HTM and ETM. The optimized processing route under ambient conditions yielded a PCE of 11 %. A low temperature processing was adopted by You et al. [24] to deposit inverted ITO/PEDOT:PSS/CH_3_NH_3_PbI_3−*x*_Cl_*x*_/PCB/Al on a flexible polyethylene terephthalate (PET) substrate, where a PCE of 9.2 % was achieved. In the aforementioned architecture, ITO stands for indium tin oxide, PEDOT:PSS for poly(3,4-ethylenedioxythiophene) polystyrene sulfonate, and PCBM for phenyl-C61-butyric acid methyl ester. Zhou and co-workers [17] reduced the carrier recombination by solution processing under a controlled humidity and also improved the electron transport channel, using yttrium-doped compact-TiO_2_ as an ETM [17], where a PCE of 16.6 % was achieved through this combined interface engineering.

The quality and functionality of the perovskite film and the complete device strongly depends on the deposition technique and processing conditions and steps. Several deposition techniques are currently used for the fabrication of perovskite solar cells, including vapor-assisted solution-processed, vapor deposition, and one-step or two-step solution-processed techniques [17–30], while casting and solution-processed methods are prefered. Adopting a proper casting method crucially affects the film coverage, thickness, precursor conversion, structure, and charge mobility and consequently the functionality of the whole device. The deposition approach in the present work is based on a sequential two-step technique (spin/spin or spin/dip) in a planar architecture. This method has been used in mesoporous structures as well [16, 27–29]. The two-step deposition of perovskite layer from its precursors was first proposed by Burschk et al. [27] for preparation of perovskite pigments embedded in mesoporous TiO_2_ film. It was found that the two-step sequential deposition allows efficient chemical conversion and a better control over the crystal size and morphology than is possible with single-step co-deposition of the precursors. Sequential deposition of perovskite precursors also prevents accelerated crystallization of perovskite as typically occurs in one-step co-deposition approach, improving reproducibility of the films and the performance of the cell [27]. Burschk and co-workers also reported a different orientation of sequential deposition of lead halide crystals induced by anatase scaffold. However, depositing on a flat surface (as required for planar structure) showed a preferential orientation along the *c* axis. They realized that on a flat substrate, conversion of PbX_2_ (lead halide) to perovskite upon exposure to CH_3_NH_3_I (MAI) may be incomplete, as a large amount of unconverted lead halide was recognized in the XRD patterns.

The effect of solvent evaporation rate or solvent volatility on chemical conversion and crystallization of perovskites is another open question in this field. Barrows et al. [21] employed nonvolatile (high boiling point) organic solvents to process the perovskite precursor solutions to delay the crystallization of spray-on perovskite films. They showed that using nonvolatile solvents, such as dimethylformamide (DMF) and dimethylsulfoxide (DMSO) can cause a large variation in the film local thickness, shrinkage, and dewetting due to prolonged drying times. They attempted to address the above-mentioned issues by spraying the solution onto a substrate held at an elevated temperature, followed by a thermal process to remove the excess solvent, organic, and halide materials. However, we believe that spraying on a hot substrate causes rapid drying of droplets individually, resulting in incomplete merging and the formation of pinholes on the ensuing spray-on thin film [31–33].

**Fig. 1:**
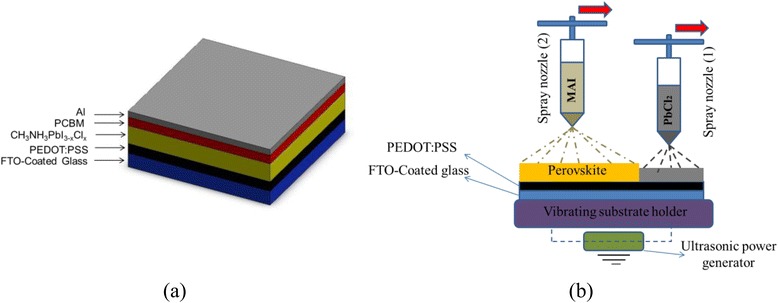
Schematic illustration of **a** adopted device architecture in this work (inverted planar heterojunction perovskite solar cell) and **b** the experimental procedure for the fabrication of perovskite layer using the 2S-SVASC method*.* In part (**b**), PbCl_2_ solution is sprayed first and then the MAI solution is sprayed atop the PbCl_2_ layer, using a second sprayer

In pursuit for finding viable solutions to the above-mentioned drawbacks and given that the development of scalable techniques [31–36] is one of the main concerns for the future of the emerging PV industry, here we present a modified scalable spray-coating technique to fabricate perovskite thin films with high coverage and uniformity. We have recently developed several modified coating techniques, i.e., ultrasonic substrate vibration-assisted spray coating (SVASC) [35], ultrasonic substrate vibration-assisted drop-casting (SVADC) [32], and spin coating followed by substrate vibration post treatment (SVPT) [37], by which imposing an ultrasonic vibration with controlled power and duration on the substrate can lead to the formation of a stable wet film and an intact and uniform thin solid film after drying. The energy imparted by an ultrasonic transducer, attached beneath the substrate, to the liquid droplets or a wet film improves substrate wetting and uniformity of the film. This energy also prevents sintering of precipitating crystallites which typically occurs due to the thermal effects or the high surface activity of ultra-fine crystals. Thus, in the current work, the concept of the imposed substrate vibration for controlling the film characteristics is applied on perovskite layers through a method termed here as the two-step sequential substrate vibration-assisted spray coating (2S-SVASC). As far as large-scale fabrication of perovskite solar cells is concerned, the modified spray method is fast, rather low-cost, and compatible with roll-to-roll fabrication process. This is in contrast to spin coating, which despite its success in the lab for the fabrication of uniform thin films, is essentially a bench-scale and batch process and cannot be scaled up. In the case of deposition of perovskites, it is expected that the ultrasonic substrate vibration would enhance the quality of the thin film through an improvement in droplet spreading and surface wetting, prohibition of the aggregation of crystallites, crystal growth with a symmetric orientation, acceleration in solvent evaporation from a stable wet film, and enhanced mass transfer and better mixing of perovskite reagents. The prepared perovskite thin films are incorporated into an inverted structure planar perovskite solar cell, in which PCBM and PEDOT:PSS are, respectively, utilized as ETM and HTM, and a single layer of Al serves as the back contact. Figure 1 presents the schematic configuration of the fabricated device and details of the experimental setup. Surface morphology and coverage and chemical composition and functionality of the active layer and the PCE of the obtained devices using various methods are compared demonstrating the merit of the 2S-SVASC method.

## Methods

The following reagents were purchased from Sigma-Aldrich, USA: DMF, ethanol, isopropyl-alcohol (IPA), chlorobenzene, DMSO, lead chloride powder, and PCBM. Methylammonium iodide (MAI) was purchased from Xi’an Polymer Light Technology, China. The high-conductive grade PEDOT:PSS in the form of 1.3 % aqueous solution was supplied by Heraeus, USA. Fluorine-doped tin oxide (FTO) conductive glass substrates (10 × 10 × 2 mm, 8 Ohm cm^−2^) were purchased from Shilpa Enterprises, India.

To prepare perovskite reagents, MAI was dissolved in IPA to obtain a solution with a concentration of 0.1 mg mL^−1^ and was heated to 70 °C before spraying onto the substrate. PbCl_2_ was dissolved in a 3:1 volume ratio of the mixture of DMSO and DMF. The resultant solution with a concentration of 0.26 mg mL^−1^ was then sonicated in a supersonic bath at 80 °C for several hours before spraying.

To prepare the PEDOT:PSS solution, IPA was added to the as-received PEDOT:PSS aqueous solution with the volume ratio of 3:1, respectively. The resultant mixture was sonicated for 1 h in ambient condition. The precursor solution was filtered by a 0.45-μm PTEF and then heated to 65 °C before spin casting. The FTO-coated glass substrates were cleaned in an ultrasonic bath by detergent, IPA, and deionized water, for 15 min, then dried in a vacuum furnace and cleaned in an ultraviolet cleaner for 15 min. The PCBM in chlorobenzene solution was prepared at concentration of 50 mg mL^−1^, stirred and heated overnight at 70 °C, and filtered through a 0.45-mm PTFE filter before casting.

For the fabrication of PEDOT:PSS thin film, a small amount of the precursor solution was placed on a clean FTO-coated glass dropwise and spun at 3000 rpm for 30 s. Before post heat treatment, the as-spun wet film was cured on an ultrasonically vibrating transducer (DMHCN-150105-A02, Clangsonic, China) at ultrasonic power of 5 W and frequency of 40 kHz for 15 s, based on the substrate vibration-assisted post treatment method (SVPT) [37]. The spun/post-vibrated PEDOT:PSS thin films were then annealed on a hot plate at 120 °C for 35 min. Both precursor and substrate were heated to 70 °C before being subjected to spin casting. Table 1 shows the effect of substrate vibration on spun-on PEDOT:PSS films to be discussed later.

**Table 1:** Characteristics of PEDOT:PSS buffer layers prepared by regular spin coating and spin coating followed by substrate vibration post treatment (SVPT)

Sample no.	Vibration power (W)	Roughness (nm)	Thickness (nm)	Coverage (%)	Conductivity (S/cm)
1	5	21	45	96	565
2	0	70	140	68	82.5

The mixed-halide perovskite active layer (CH_3_NH_3_PbI_3−*x*_Cl_*x*_) was made atop the spun-on PEDOT:PSS layer using the 2S-SVASC method as explained in the introduction and depicted in Fig. 1. The core of this experimental set up is a spray-coating machine (Holmarc, Opto-Mechatronics Pvt. Ltd., Model HO-TH-04, India), equipped with two syringe pumps connected to capillary tubes for injection of precursor solutions using air-assist atomization. The atomizing gas at a pressure of 0.3 MPa was provided by an oil-free air-compressor. To fabricate the active layer thin film, mixed-halide perovskite, PbCl_2_, and MAI solutions were pre-loaded into the syringe pumps and injected sequentially through capillaries (0.6-mm ID). The same ultrasonic transducer used for post treatment of spun-on PEDOT:PSS was employed as the substrate holder and the ultrasonic vibration provider during the spray-coating process. The temperature of the syringe pumps and the surface of ultrasonic transducer were both controlled by a temperature controller. The speed and distance of nozzle tip to the substrate, the flow rate of each solution, the time interval between two sprays, and the process duration were controlled by software. The operating conditions and some characteristics of perovskite films made by 2S-SVASC method are listed in Tables 2 and 3. The process variables listed in the tables have been determined to provide a 3:1 molar ratio of MAI to PbCl_2_ reagents.

**Table 2:** Operating conditions and characteristics of perovskite films fabricated by single-step and sequential 2S-SVASC spray methods

Run	TIS (min)	Substrate temp. (°C)	Vibration power (W)	Roughness (nm)	Surface coverage (%)
1	0.17	70	5	230	61
2	5	70	5	190	54
3	15	70	5	130	59
4	0.17	120	5	250	72
5	5	120	5	61	91
6	15	120	5	52	96
7	15	120	0	420	53
8	15	120	10	240	31
9^m^*	–	120	0	170	35
10^m^*	–	120	5	66	82

*One-step (co-deposition) spraying; TIS it the time internal between sequential sprays

**Table 3:** Operating conditions of spraying steps used for the fabrication of perovskite thin films by 2S-SVASC

Reagent	Solvent	Nozzle tip speed (mm/s)	No. of spray passes	Spray flow rate (μL/min)	Concentration (g/mL)
PbCl_2_	DMSO/DMF (vol. ratio: 3/1)	50	1	200	0.26
MAI	IPA	5	2	400	0.01

In order to elucidate the importance of the sequential spraying, we performed two experimental runs (9^m^ and 10^m^) based on the conventional one-step spraying, with and without imposed ultrasonic vibration on the substrate. In this case, the reagent solutions (MAI in IPA and PbCl_2_ in DMSO + DMF, at a 3:1 ratio of MAI to PbCl_2_) were mixed at 70 °C and filtrated through a 30-μm PTFE filter under nitrogen atmosphere in a glovebox. The filtrate was loaded into a syringe pump and sprayed onto the substrate, while the substrate and solution were both kept at a 70 °C. The structural characteristics and performance of the prepared thin films were compared with the perovskite thin films prepared via the 2S-SVASC method. As mentioned earlier, a 3:1 volume ratio of DMSO/DMF was used to prepare the lead halide precursor. DMSO makes a strong coordination with PbCl_2_ owing to its –O– groups. A good coordination between the solvent and solute causes a uniform deposition and high surface coverage [20]. However, complete removal of DMSO needs prolonged annealing times at elevated temperatures, making the perovskite susceptible to decomposition. On the other hand, interaction between DMSO and PbCl_2_ causes retarded deposition due to formation of strong DMSO–Pb– complexes [22]. Using pure DMF also leads to a very fast chemical reaction in a few seconds after contact between reagents has been made. This immediate conversion results in rapid and irregular crystallization and unfavorable chemical conversion, coverage, and morphology. Addition of DMF to DMSO optimizes the reaction between precursors and solvent volatility and evaporation rate. We examined different ratios of DMSO/DMF. It was observed that for the conditions of these experiments, the best film quality and coverage is achieved when a 3:1 volume ratio of DMSO/DMF is employed as the solvent for PbCl_2_.

PCBM precursor was spray-deposited atop the glass/FTO/PEDOT:PSS/perovskite stacked films placed on an ultrasonic transducer, working at 5 W vibration power, at 80 °C. The PCBM precursor solution was loaded into a syringe and sprayed through a 0.8-mm capillary, while the nozzle tip was kept at a 4-cm distance from the substrate. The as-sprayed PCBM thin film was then annealed at 70 °C for 90 min on an electrical pan under the N_2_ atmosphere in a glovebox. A 100-nm back contact (Al) was thermally evaporated outside of the glovebox. Devices were finally covered by UV-cleaned bare glasses (1 cm × 1 cm × 1 mm) and sealed by UV-epoxy paste before the current-voltage characterization.

Scanning electron microscopy (SEM, Hitachi, Model S-3400N, Japan) was used to study the surface and cross-sectional morphology of the fabricated thin films and device. Surface profiles, 2D and 3D images, and the thickness of thin films were obtained by a confocal laser scanning microscope (CLSM, Zeiss, model LMS700, Germany). The optical mode of this machine was utilized to observe the surface topography. Electrical conductivity of spun-on PEDOT:PSS films was measured via the 4-point probe measurement technique, along a 16-mm straight line on each sample. Light absorbance of perovskite and transmittance of PEDOT:PSS films were measured by a UV-visible absorption/transmission instrument (Shimadzu UV-3101PC UV-Vis-NIR spectrophotometer). The device current-voltage curves and PCE were obtained by Labview software and a source meter (National Instruments, model NI PXI-1033, TX, USA). Coverage values were measured visually through a simple color contrast difference analysis, using ImageJ-1.49 software. The device incident photon-to-charge carrier efficiency (IPCE) spectra were obtained by a quantum efficiency measurement system (QEPVSI-b, Newport, USA), showing the external quantum efficiency (EQE) of the device.

## Results and Discussion

**Fig. 2:**
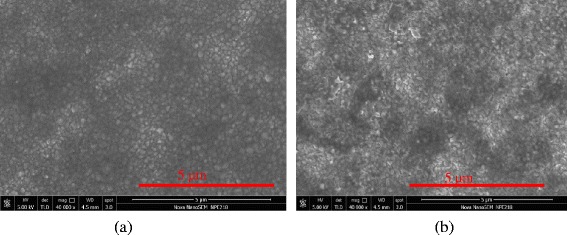
SEM images of PEDOT: PSS thin films, prepared by spin coating: **a** as-spun wet film vibrated on an ultrasonic transducer, using the SVPT method, operating at 5 W for 15 s, before thermal processing. **b** The wet film was dried and cured naturally. The wet films were subjected to heat treatment at 120 °C for 35 min

Table 1 lists the characteristics, and Fig. 2 shows topography of PEDOT: PSS films, prepared by spin coating followed by ultrasonic substrate vibration post treatment (SVPT) [37] and regular spin coating. The surface quality and the electrical properties are significantly improved when a spun wet film is treated by ultrasonic vibration using SVPT. Table 1 and Fig. 2 show that the SVPT significantly enhances the film conductivity, surface coverage, and film uniformity (fewer pinholes). The effect of the imposed substrate vibration on the nanostructure of spun-on PEDOT:PSS thin films has been studied in detail in our previous work [37]. In brief, imposed vibration results in the detachment of conducting PEDOT from insulator PSS and realignment of PEDOT chains, making a continuous conducting network, thus improving conductivity. Also, under controlled conditions, imposing low power vibration can promote mixing and stability of the film [36]. Increased evaporation as a result of vibration can also cure the film faster and before pinholes formation.

**Fig. 3:**
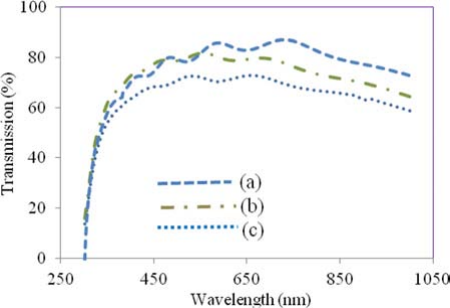
UV-Vis transmission spectra of *(a)* FTO-coated glass, *(b)* PEDOT: PSS thin film fabricated on FTO-coated glass by spin coating followed by substrate vibration post treatment (SVPT), and *(c)* PEDOT: PSS thin film fabricated on FTO-coated glass by regular spin coating

Figure 3 illustrates the UV-Vis transmission spectra of FTO-coated glass, and PEDOT:PSS thin films fabricated on FTO-coated glass by spin coating followed by SVPT and by regular spin coating. It is observed that the SVPT improves the light transmission and transparency of PEDOT:PSS thin film. This improvement in transparency is due to a better uniformity and a thinner and smoother film formed (c.f. Table 1) resulting in better light transmission, making the film suitable to be utilized as the HTM in planar perovskite solar cells, fabricated in this work.

**Fig. 4:**
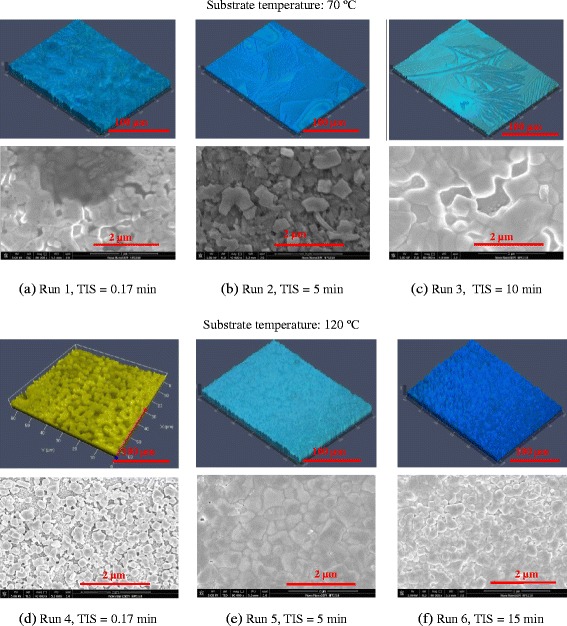
Effect of the substrate temperature and the time interval between sprays (TIS) on the perovskite film topography: CLSM (*top rows*) and SEM (*bottom rows*) images of the perovskite thin films prepared by the 2S-SVASC technique (runs 1–6). Images (**a**), (**b**), and (**c**) correspond to the films fabricated at substrate temperature of 70 ºC, and (**d**), (**e**), and (**f**) correspond to the films fabricated at substrate temperature of 120 ºC

In the process of the fabrication of perovskite solar cells, the perovskite active layer is made by spray coating atop the spun-on PEDOT:PSS film (c.f. Fig. 1). Figure 4 displays a series of optical and SEM images of the perovskite layers showing the effect of the substrate temperature and time interval between two spraying stages (TIS) of the perovskite precursors on topography of the perovskite films, while the other process parameters are kept constant (runs 1–6 of Table 2). According to the surface topography and morphology images (Fig. 4) and the roughness data in Table 2, the coverage, morphology, and quality of perovskite thin films are substantially affected by the substrate temperature and the TIS. The effects of the substrate temperature on the film structure of spray-on PEDOT: PSS films were investigated in our previous reports [33, 35, 36], where it was inferred that spraying on a high temperature substrate results in the formation of numerous defects and pinholes on the PEDOT:PSS film due to the fast evaporation, bubble formation, and poor droplet spreading and coalescence. Therefore, the two-step sequential substrate vibration-assisted spray coating (2S-SVASC) was employed for the fabrication of perovskite films to suppress the above-mentioned drawbacks. As illustrated in Fig. 4, spraying onto a low temperature substrate (70 °C) results in irregularities in the film with multiple domains or features (Fig. 4a), changing to a ribbon- or filament-like structure (Fig. 4b, c), with increasing TIS. Sequential spraying on a high temperature substrate (120 °C) leads to spiky and grainy features uniformly distributed, where increasing the TIS leads to decreasing the size and increasing the density of the grains (Fig. 4d–f). In most cases (except for TIS = 0.17 min), the roughness of the spray-on perovskite films is higher at the low substrate temperature of 70 °C, compared to that of the high substrate temperature of 120 °C. The large roughness can be attributed to the ribbon- or filament-like features. Such crystallites present colorless or pale yellow color, a signature of monohydrated and di-hydrated perovskite phases [38, 39]. In the following, we explain the effect of the hydration on perovskite morphology and crystal growth and the effect of the substrate temperature on this phenomenon. Incorporation of water into the perovskite lattice results in disordering of 3D networks to 1D (needle-type) and 0D (amorphous) features. We suggest a series of chemical reactions for the hydration of a mixed-halide (halide = Cl and I) perovskite as follows:

R1 $$ \mathsf{4}\left[{\mathsf{CH}}_{\mathsf{3}}{\mathsf{NH}}_{\mathsf{3}}{\mathsf{PbI}}_{\mathsf{3}-\mathit{\mathsf{x}}}{\mathsf{Cl}}_{\mathit{\mathsf{x}}}\right]+\mathsf{4}{\mathsf{H}}_{\mathsf{2}}\mathsf{O}\leftrightarrow \mathsf{4}\left[{\mathsf{CH}}_{\mathsf{3}}{\mathsf{NH}}_{\mathsf{3}}{\mathsf{PbI}}_{\mathsf{3}-\mathit{\mathsf{x}}}{\mathsf{Cl}}_{\mathit{\mathsf{x}}}.{\mathsf{H}}_{\mathsf{2}}\mathsf{O}\right]\leftrightarrow {\left({\mathsf{CH}}_{\mathsf{3}\ }{\mathsf{NH}}_{\mathsf{3}}\right)}_{\mathsf{4}}{\mathsf{PbI}}_{\mathsf{6}-\mathsf{2}\mathit{\mathsf{x}}}{\mathsf{Cl}}_{\mathsf{2}\mathit{\mathsf{x}}}.\mathsf{2}{\mathsf{H}}_{\mathsf{2}}\mathsf{O}+\mathsf{2}{\mathsf{H}}_{\mathsf{2}}\mathsf{O}+\left(\mathsf{3}-\mathit{\mathsf{x}}\right){\mathsf{PbI}}_{\mathsf{2}}+\mathit{\mathsf{x}}{\mathsf{PbCl}}_{\mathsf{2}} $$

The monohydrate $\left({\mathsf{CH}}_{\mathsf{3}}{\mathsf{NH}}_{\mathsf{3}}{\mathsf{PbI}}_{\mathsf{3}-\mathit{\mathsf{x}}}{\mathsf{Cl}}_{\mathit{\mathsf{x}}}.{\mathsf{H}}_{\mathsf{2}}\mathsf{O}\right)$ forms a metastable thin and colorless needle-type feature [38]. In this compound, the 1D isolated [PbICl_2_]^−^ double chains, forming the linked [PbICl_2_]^4−^ octahedron wide ribbons, are responsible for the needle or fibrous configuration [38, 39]. In mixed-halide perovskite formulation, the isolated [PbICl_2_]^−^ chains are balanced by [CH_3_NH_3_]^+^ cations, and the H_2_O molecules located between [PbICl_2_]^−^ double chains increase the stability, owing to the symmetrical hydrogen bonds between H_2_O and methyl ammonia. This is a reversible process occurring during film drying in dark and vacuum [38]. The di-hydrated perovskite ${\left({\mathsf{CH}}_{\mathsf{3}\ }{\mathsf{NH}}_{\mathsf{3}}\right)}_{\mathsf{4}}{\mathsf{PbI}}_{\mathsf{6}-\mathsf{2}\mathit{\mathsf{x}}}{\mathsf{Cl}}_{\mathsf{2}\mathit{\mathsf{x}}}.\mathsf{2}{\mathsf{H}}_{\mathsf{2}}\mathsf{O}$, however, forms a 0D matrix of isolated [PbICl_2_]^4−^ octahedral anions surrounded by neutralizing [CH_3_NH_3_]^+^ cations, which form an irregular lattice. In highly prolonged exposure to humid media, an irreversible reaction occurs as follows, which is associated with discoloring to an opaque yellow color, a signature of PbI_2_ crystals [38, 39].

R2 $$ {\left({\mathsf{CH}}_{\mathsf{3}\ }{\mathsf{NH}}_{\mathsf{3}}\right)}_{\mathsf{4}}{\mathsf{PbI}}_{\mathsf{6}-\mathsf{2}\mathit{\mathsf{x}}}{\mathsf{Cl}}_{\mathsf{2}\mathit{\mathsf{x}}}.\mathsf{2}{\mathsf{H}}_{\mathsf{2}}\mathsf{O}\to \mathsf{2}{\mathsf{H}}_{\mathsf{2}}\mathsf{O}+\left(\mathsf{3}-\mathit{\mathsf{x}}\right){\mathsf{PbI}}_{\mathsf{2}}+\mathsf{4}{\mathsf{CH}}_{\mathsf{3}\ }{\mathsf{NH}}_{\mathsf{3}}\mathsf{C}\mathsf{l}\kern0.75em \uparrow (g)+\left(x-\mathsf{2}\right){\mathsf{Cl}}_{\mathsf{2}}\uparrow (g) $$

A hydrated perovskite specimen may contain both mono- and di-hydrated phases, as well as some decomposed parts that contain PbI_2_ [38, 39]. It has been shown that the irreversible perovskite decomposition to PbI_2_ can only happen by water condensation on perovskite surface, resulted from a temperature difference between the film surface and the humid air [38]. In the current work, the substrate temperature is much higher than the ambient temperature. It is therefore assumed that condensation never occurs during deposition, but partial condensation may take place during the sample analysis. The H_2_O produced in di-hydration reaction (R1) may transform the remaining perovskite phase. Irreversible decomposition can also occur without the requirement for the excess water when ${\left({\mathsf{CH}}_{\mathsf{3}\ }{\mathsf{NH}}_{\mathsf{3}}\right)}_{\mathsf{4}}{\mathsf{PbI}}_{\mathsf{6}-\mathsf{2}x}{\mathsf{Cl}}_{\mathsf{2}x}.\mathsf{2}{\mathsf{H}}_{\mathsf{2}}\mathsf{O}$ is exposed to light [38, 39]. On the basis of this simple theory, we can recognize that the thin film made at run 1 has undergone a complete hydration path based on reactions R1 and R2, leaving different (rod-like, grainy, and amorphous) regions on the surface, while in thin films formed at runs 2–3, the monohydrated phase occupies a bigger part. This interesting evolution resulted from the effect of different TISs. We reiterate that in our sequential method, the PbCl_2_ precursor solution is sprayed in the first step and the MAI solution is sprayed in the second step. Contribution of TIS to the hydration process can be elucidated as follows: a longer TIS allows more depletion of DMSO/DMF from PbCl_2_ precursor solution. Both DMSO and DMF are strongly polar, increasing the surface activity and tendency for water absorption. On the other hand, a different feature and trend is observed when the substrate is kept at 120 °C (runs 4–6). The films are seemingly composed of grainy or spiky crystallites, whose size and density significantly changes by the TIS. We assume that the thin films deposited on high temperature substrates have experienced a reversible hydration, but the absorbed H_2_O is removed instantly, due to the very high substrate temperature (120 °C). On the other hand, the hydration is an exothermic (thermo-phobic) reaction which can be suppressed in high temperature conditions. It is, therefore, proposed that the perovskite thin films deposited on a hot substrate are relatively stable against hydration during the deposition process. The change in density and crystal size due to a change in the TIS can be again due to the effect of the solvent content. As mentioned above, a longer TIS (especially with high temperature substrate) results in less DMSO/DMF and more IPA in the wet precursors on the substrate and therefore a decrease in the normal boiling point and an increase in wettability [36]. Reduction of the normal boiling point of the solvents causes faster and more uniform crystallization, while better wettability favors droplet spreading and coalescence and surface coverage, consequently [22, 40]. It is worth mentioning that unlike the literature reports, we recognized that no crystal sintering occurs at high temperature deposition. We ascribe this advantage to the imposed ultrasonic vibration prohibiting crystals aggregation [32, 35].

**Fig. 5:**
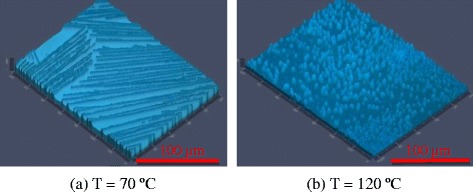
CLSM images of spray-on PbCl_2_ thin films made on ultrasonically vibrating substrates kept at **a** 70 °C and **b** 120 °C. The samples were annealed at the same temperature as the substrate temperature for 15 min. In (**a**), roughness = 159 nm and coverage = 46 %, and in (**b**), roughness = 55 nm and coverage = 68 %. The fabrication conditions of PbCl_2_ films are the same as those of runs (1–3) for (**a**) and runs (4–6) for (**b**), and TIS is irrelevant in this case. At 70 °C, PbCl_2_ film has a rode-like topography; therefore, the perovskite film made at this temperature is also rode-like, while at 120 °C, the PbCl_2_ film is grainy and so is the ensuing perovskite film (c.f. Fig. 4)

In sequential deposition, the form and quality of the precursor PbCl_2_ thin film strikingly determines the final form and quality of the perovskite thin film. Burschk et al. [27] showed that in an attentively controlled sequential deposition of precursors, the final perovskite crystals will be similar to those of the first deposited precursor, lead halide (PbX_2_). This preserving of size and morphology is only accessible with sequential deposition into a porous template, such as mesoporous TiO_2_. But in a flat substrate, as in the planar architecture, the deposited PbCl_2_ acts as a template as well as a reagent for the formation of perovskite. We observed that a smaller, denser, and more uniform PbCl_2_ thin film helps the creation of a more uniform, smooth, and intact perovskite thin film. Following the above discussion on the importance of the morphology of the PbCl_2_ film on the final morphology of the perovskite film, we studied the structure of PbCl_2_ thin films sprayed on an ultrasonically vibrating substrate in Fig. 5 and its relation to the characteristics of the perovskite films. The PbCl_2_ thin films shown in Fig. 5 were made by spraying the PbCl_2_ precursor, while the substrate was kept at 70 °C (Fig. 5a) and 120 °C (Fig. 5b). Each sample was annealed for 15 min at the same temperature as that of the substrate in order to simulate the formation of PbCl_2_ layer of perovskite films in runs 3 and 6, respectively. The PbCl_2_ deposited on low temperature substrate (Fig. 5a) experiences a prolonged solvent evaporation, thus promoting crystal growth and aggregation [22, 40]. The 1D orientation of crystal growth in Fig. 5a is perhaps due to the dissociation of interlinked Cl–Pb–Cl planes by imposed ultrasonic vibration. Deposition of PbCl_2_ on the high temperature substrate (Fig. 5b), on the other hand, occurs following an accelerated evaporation, rapid nucleation, and a fast crystal growth, creating small size and narrow distributed crystallites [22, 40]. High surface coverage and low roughness are achieved owing to imposed ultrasonic vibration during nucleation and crystal growth.

Having discussed the formation of the PbCl_2_ films (Fig. 5), its effect on the ensuing perovskite films (Fig. 4) may be rationalized as follows: the main driving force for initiation of the perovskite formation reaction is the difference between the bulk lattice energies of the two reagents, i.e., PbCl_2_ and MAI. The PbCl_2_ crystal lattice serves as a template for the formation of the target compound, i.e., perovskite [38, 39]. The sprayed MAI (NH_3_CH_3_^+^) on PbCl_2_ is likely to be shaped by the large matrix of PbCl_2_ consisting of repeating Cl–Pb–Cl planes. The configuration of the organic cations between these layers is controlled by the strong inter-layer chemical bonding and the weak van der Waals forces between them. This scenario is well consistent with the condition of runs 2 and 3 and particularly with the condition of runs 5 and 6, in which PbCl_2_ crystals are completely formed (long TIS) before the MAI solution is sprayed atop. The absence of voids in the perovskite films in runs 5 and 6, compared to the precursor PbCl_2_ thin film, implies reorganization of PbCl_2_ and MAI due to the intensive diffusion during the film growth [5].

**Fig. 6:**
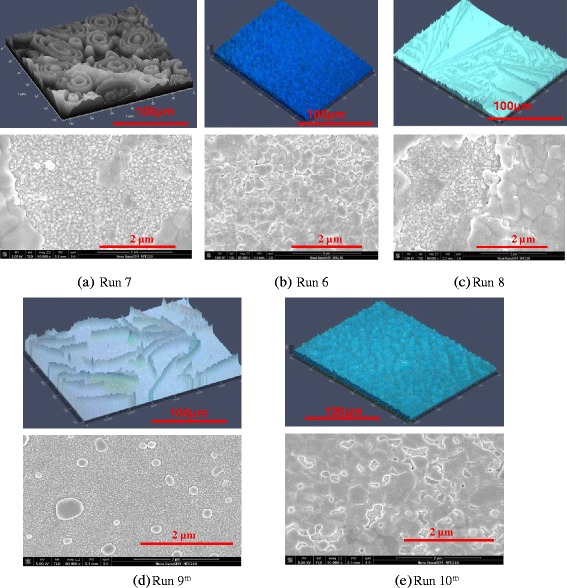
CLSM images (*above*) and SEM images (*below*) of perovskite thin films prepared by **a** sequential spray coating on stationery substrate (run 7), **b** 2S-SVASC at a power of 5 W (run 6), **c** 2S-SVASC at a power of 10 W (run 8), **d** co-spray-on stationary substrate (run 9^m^), and (**e**) co-spray-on a vibrating substrate (run 10^m^). Run 9^m^ has been performed by co-spray using the conditions of run 7, performed by sequential sprays, and run 10^m^ has been performed by co-spray, using the conditions of run 6, performed by sequential sprays. The background small grain film detected in SEM images is PEDOT: PSS

The main advantage of our 2S-SVASC deposition approach compared with the state-of-the-art methods for deposition of perovskite films is the application of the imposed ultrasonic vibration on the substrate and its combination with spray coating, which makes it a controllable scalable method. To study the effect of power (amplitude) of the imposed vibration, Fig. 6 presents the topography and morphology of perovskite thin films prepared in runs 6, 7, and 8. In Fig. 6a, the concentric circles grown on a rough surface illustrate impinging and drying of individual droplets on a non-vibrating or stationary substrate, indicating poor droplet merging, lack of the formation of an intact film, and insufficient contact between the two precursors. In Fig. 6c, outspread crystal growth implies the rupture of precursor wet film by intensive vibration (10 W), while in run 6 (Fig. 6b) with the same condition, a mild vibration power (5 W) results in uniform crystal size distribution and high surface coverage as discussed before. These results suggest that in a given frequency, there is a threshold vibration power beyond which the ultrasonic vibration causes film rupture and dewetting. This threshold depends on the physical and interfacial properties of the precursor solution [36] and needs to be addressed for each individual system.

In order to examine the merit of the two-step sequential method, in which two perovskite precursors are sequentially sprayed, perovskite films were also fabricated by one-step co-spray deposition of a mixture of the two precursor solutions on stationary (run 9^m^) and vibrating substrates (run 10^m^). Figure 6d shows that a rough, non-uniform perovskite layer with poor coverage is formed by the co-spray method on a stationary substrate (run 9^m^), somewhat similar to its sequentially made counterpart (run 7 of Fig. 4), although the coverage of run 7 is higher. When the substrate is vibrated (Fig. 6e), the film characteristics are improved, and the film resembles its sequentially fabricated counterpart (run 6), although the coverage of co-sprayed sample (c.f. SEM of run 10^m^) is lower than the sample sequentially sprayed (c.f. SEM of run 6). This is because in co-spraying approach, due to the strong interaction between DMSO and IPA, the reagents partially precipitate upon mixing. This early crystallization disturbs the balance of molar ratio between PbCl_2_ and MAI reagents in the liquid phase. Therefore, the precursor solution after mixing consists of a sintered solid phase and a dilute liquid phase, which should be essentially filtrated before spraying. The resultant filtrate will be a dilute bright yellow solution, leaving behind a thin layer with low coverage due to the lack of sufficient reagents. Therefore, a combination of sequential spraying and substrate vibration through the 2S-SVASC renders the best perovskite film characteristics.

**Fig. 7:**
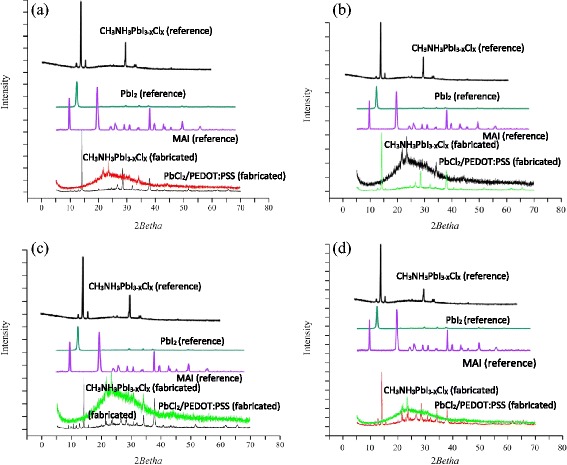
X-ray diffraction spectra of PbCl_2_ (before deposition of MAI precursor) and perovskite thin films prepared at selected runs as listed in Table 2: **a** run 5, **b** run 6, **c** run 3, and **d** run 10^m^

**Fig. 8:**
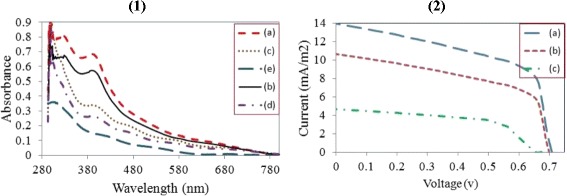
*(1)* Light absorbance of perovskite thin films prepared at: *(a)* run 6, *(b)* run 5, *(c)* run 10^m^, *(d)* run 3, and *(e)* PbCl_2_ thin film sprayed on a vibrating substrate, at substrate temperature = 120 °C. *(2)* Current density-voltage curves of planar perovskite solar cells, where the perovskite layer is fabricated at conditions of *(a)* run 6, *(b)* run 5, and *(c)* run 10^m^

Figure 7 depicts the XRD crystallography patterns of selected films (runs 3, 5, 6, and 10^m^) showing the peaks of PbCl_2_ and their corresponding perovskite thin films. The reference graphs were adopted from the literature [21, 25, 38, 41, 42]. The XRD patterns of PbCl_2_ consist of two sharp peaks superimposed on a broad scattering background related to the underneath PEDOT:PSS film. Comparing with the literature, the best perovskite thin films were obtained in runs 5 and 6 (Fig. 7a, b) with major peaks at 14.2° (110), 28.4° (220), and 43.8° (330), indicating the tetragonal structure of CH_3_NH_3_PbI_3−*x*_Cl_*x*_ [21, 25, 38]. The narrow peaks suggest that the film has a long range crystalline domain (higher than 200 nm) [38], highly oriented in the a-axis. In contrast to the single-halide structure, this iodide-chloride mixed formulation is rather stable, and, therefore, amenable with ambient processing. The absence of the PbCl_2_ peaks in perovskite patterns implies a complete transformation of PbCl_2_ precursor, and the absence of typical signals of PbI_2_ at 12.6° indicates that the perovskite film is not irreversibly degraded. The other extra peaks in the perovskite XRD patterns are related to the excess MAI. Note that according to Burschk et al. [27], the perovskite film sequentially deposited by spin/dip coating on flat substrates shows a large amount of the precursor lead-halide, indicating incomplete chemical reaction of perovskite precursors. Therefore, the remarkable precursor conversion achieved in this work is attributed to the combined contribution of the imposed substrate vibration and the two-step sequential spray coating. We predict a simple scenario for this significant improvement. For a continued chemical transformation of precursors at the interface of already formed perovskite CH_3_NH_3_PbI_3−*x*_Cl_*x*_ and the existing PbCl_2_ film, where there is no van der Waals gap, a rather long and effective contact time and proper mixing is required [21, 25, 27, 41], which is not fully compatible with the regular sequential methods like spin/spin and spin/dip coating. In 2S-SVASC method, the lack of sufficient mixing and contact between the precursors is compensated by the imposed ultrasonic vibration, which acts as a kinematic driving force for mixing. Figure 8c exhibits the crystallography patterns of perovskite thin film obtained by the 2S-SVASC on low temperature substrate (70 °C, run 3). The XRD patterns enable to identify the hydrated and unreacted areas as described earlier. The reflections between 8.4°–10.7° correspond to the monohydrated phase [38]. The short peak at 11.4° verifies the existence of di-hydrated phase, initiating the decomposition process according to reaction R2. The presence of the PbI_2_ signal at 12.6° in the perovskite film indicates partial decomposition and the other peaks reflect the existence of PbCl_2_ and MAI, implying an incomplete chemical conversion. It is, therefore, deduced that a perovskite thin film deposited on low temperature substrate is vulnerable against humidity and O_2_. It can be deduced that in 2S-SVASC technique, the substrate temperature should be kept as high as possible to accelerate the chemical conversion and prevent reaction with H_2_O. The XRD patterns in Fig. 7d correspond to the thin film co-sprayed on a vibrating substrate (run 10^m^). Typical CH_3_NH_3_PbI_3−*x*_Cl_*x*_ peaks are clearly recognized. Appearing of PbCl_2_ and MAI peaks suggests an uncompleted reaction and a small peak at 12.6° (PbI_2_) is an indicative of partial decomposition. The slow chemical conversion in co-spray method may be due to the early precipitation upon mixing of precursors and losing the molar balance between the reagents.

The differences in the properties and structures of perovskite thin films fabricated under various conditions are further investigated by light absorbance spectra, presented in Fig. 8(1) and IV curves are shown in Fig. 8(2). Three champion samples (prepared in runs 5, 6, and 10^m^) as well as a failed sample (run 3) and a PbCl_2_ sample (deposited on high temperature vibrating substrate) were characterized by UV-Vis spectrometer. Absorbance spectra for the optimized samples (fabricated in runs 5 and 6) demonstrate suitable light absorbing potentials over the visible to near IR wavelengths. Photocurrent generation starts at 780 nm, in accordance with the bandgap of the CH_3_NH_3_PbI_3_Cl_3−*x*_ and reaches peak values of ∼85 % in the visible range [9, 43, 44]. A slight difference in absorbance of samples prepared at runs 5 and 6 (Fig. 8(1(a), (b))) are in well accordance with our previous observations. In fact, a higher coverage, less roughness, and higher uniformity of crystalline lattice leads to better light scattering and improves the wavelength response [25, 29, 40]. The next absorbance intensity is demonstrated by the sample fabricated under substrate vibration-assisted co-spraying (run 10^m^, Fig. 8(1(c))). The relative drop of light absorbing performance might be due to the lower surface coverage and low thickness, mitigating the light absorption function. It should be noted that the dilute precursor in co-spray method leaves an ultra-thin layer with a poor photo-absorption behavior. The blue shifted onset also reflects defective surface or heterogeneous crystalline structure, consistent with previous topography, crystallography and morphology evaluations. Photo absorbance spectra of the sample prepared at run 3 (Fig. 8(1(d))) displays a sharp drop toward green range indicating the presence of either PbI_2_ or PbCl_2_ [29]. Nonetheless, a higher photo-absorption response for the sample of run 3 compared to that of the pure PbCl_2_ (Fig. 8(1(e))) indicates a partial conversion of precursor solutions to perovskite, followed by hydration and then degradation under ambient conditions [14]. Also, a drastic reduction in light absorbance in sample of run 3 compared to the other samples is the signature of surface ruptures and low coverage, as discussed earlier.

**Fig. 9:**
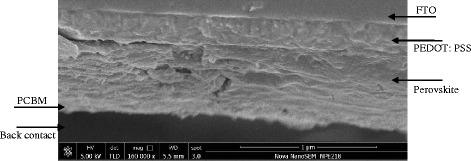
Cross sectional SEM image taken across the cleaved edge of a planar glass/FTO/PEDOT:PSS/CH_3_NH_3_I_3−*x*_Cl_*x*_/PCBM/Al device fabricated by the 2S-SVASC method

Having optimized the fabrication of the perovskite layer, a series of devices were prepared by spray deposition of PCBM atop the active layer, and thermal evaporation deposition of Al, as the back contact. In all cases, the device active area is greater than 0.3 cm^2^. Figure 8(2) presents the current density-voltage (*J*-*V*) characteristics of the fabricated planar devices employing the thin films prepared at runs 5, 6, and 10^m^. *J*-*V* curves were obtained in the range from forward (+1 V) to reverse (−1 V) bias, under AM1.5G solar irradiation with intensity of 1000 W m^−2^. Performance parameters are presented in Table 4. The maximum film coverage, minimum roughness, and the most uniform morphology was achieved by the perovskite thin film obtained at run 6; as a result, the device made based on the perovskite film of run 6 showed the highest PCE of 5.08 %. The average perovskite film thickness for this device was determined to be 460 ± 10 nm, creating a balance between the charge diffusion length, surface coverage, and the rate of recombination [42]. The relatively uniform and dense crystalline structure of perovskite in this optimized sample enhances charge generation efficiency and consequently results in better diode-like behavior of the device. In addition, an intact, smooth, and uniform perovskite layer enables sufficient adhesion with HTM and ETM, and less charge transfer resistance at the interfaces [16, 18, 20, 43]. For the mixed-halide planar structures, higher performance has been reached with smaller thickness of active layer close to 390 nm [44]. We infer that the over-growth of nanocrystals typically occurs in sequential spray, which requires further controlling over the process parameters. The relatively high fill factor attained by this sample might be originated from the better surface uniformity and crystalline morphology of the active layer compared to the other samples. The high short-circuit current (Fig. 8(2(a))) compared to the other samples unveils that the device benefits from a longer lifetime and more efficient transport of charge carriers in the CH_3_NH_3_PbI_3−*x*_Cl_*x*_ planar junction. The second device fabricated using the sample of run 5, generated PCE of 3.54 %. The average thickness of this sample was measured to be 590 ± 10 nm. The performance drop with respect to the first case is attributed to the wider size distribution of the nanocrystals, resulting in further recombination, insufficient charge transport through the active layer, and lower short-circuit current and fill factor, consequently. For the sake of comparison, a device was also fabricated incorporating the co-sprayed perovskite film based on run 10^m^ (substrate vibration-assisted co-spray). The average thickness of this perovskite thin film was determined to be 230 ± 10 nm. The device performance is significantly inferior to that of the devices made using the optimized perovskite thin films (runs 5 and 6 made by 2S-SVASC technique), as a result of a decreased short circuit current and open circuit voltage. Back to the topography image of run 10^m^ (c.f. Fig. 6b), isolation of perovskite crystals impede the charge carrier injection to the conductive layers, thus increasing the charge recombination. The small voids on the surface result in short circuits between the electrodes, decreased shunt resistance, and reduced device PCE. The considerable drop in *Jsc* of the co-sprayed device (run 10^m^) is perhaps due to the low thickness of the active layer and consequently the short diffusion length and poor photon harvesting efficiency [1, 19, 21, 22, 44, 45]. The optimization of the substrate vibration-assisted co-spray process is currently under further investigation, due to its simplicity and compatibility with large-scale fabrication of solution-processed solar cells. Figure 9 displays the SEM image of the cross section of a typical device, showing the smoothness, uniformity, and good contact between the stacked layers.

**Table 4:** Performance parameters of the solar cells derived from *J*-*V* curves obtained in the range from forward (+1 V) to reverse (−1 V) bias, under AM1.5G solar irradiation with intensity of 1000 W m^−2^

Active layer	*V*_oc_ (v)	*J*_sc_ (mA/cm^2^)	Fill factor (%)	PCE (%)
Run 6	0.74	14.26	52	5.08
Run 5	0.69	9.93	46	3.54
Run 10^m^	0.65	4.77	42	2.34

**Fig. 10:**
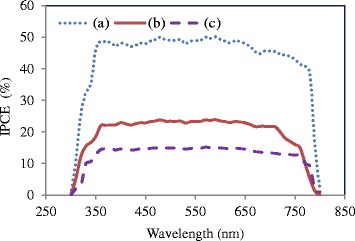
Incident photon to current efficiency (IPCE) in the form of external quantum efficiency (EQE) of the perovskite solar devices, where the active layers are fabricated at conditions of *(a)* run 6, *(b)* run 5, and *(c)* run 10^m^

The incident photon to current efficiency (IPCE) of the representative devices in the form of the external quantum efficiency (EQE) are shown in Fig. 10. The IPCE demonstrates the potential of charge generation in active layer as well as indicates the charge transport, charge collection, and mechanism of charge loss from the active layer toward the electrodes [29, 43–47]. The champion device shows the maximum IPCE (Fig. 10a) of 40–50 % in a wide range of wavelengths (340–790 nm). The rather low IPCE in this simple cell structure compared to the state-of-the-art devices, e.g., [21], may be due to the imperfect structure of spray-deposited PCBM, leading to insufficient charge collection at the electrode/ETM and perovskite/ETM interfaces. It is also worth noting that the similar inverted structures with Al electrode incorporate an ultra-thin layer of Ca below Al to overcome the work function mismatch at the PCBM/metal contact junction [21, 43–47]. Application of Ca, however, imposes extra fabrication costs and is not compatible with large-scale fabrication of solar cells. In this work, Ca layer was eliminated due to difficulties associated with thermal evaporation of corrosive Ca outside of glovebox. Inclusion of Ca and detailed optimization of PCBM thickness [48] could further increase the device efficiency, topics which are currently under investigation but are beyond the scope of this fundamental work, which aimed to develop a scalable method for the fabrication of the active layer.

## Conclusions

Optimized mixed-halide perovskite layers were successfully fabricated using a novel scalable technique termed as two-step sequential substrate vibration-assisted spray coating (2S-SVASC). The perovskite layers were further embedded in a simple inverted planar heterojunction solar cell device structure to examine and demonstrate the effectiveness of the developed scalable fabrication technique. The optimization process revealed that the performance of the perovskite films prepared by the 2S-SVASC method strongly depends on the substrate temperature, substrate vibration power, and time interval between the two sequential sprays delivering the perovskite precursors to the substrate. In the optimized experimental conditions (run 6, substrate temperature = 120 °C, substrate vibration power = 5 W, and the time interval between spraying perovskite precursors = 15 min), the application of the 2S-SVASC guarantees the formation of a perovskite film with minimum density of pinholes, almost 100 % surface coverage and sufficient chemical conversion of perovskite precursors to perovskite crystals, resulting in a device power conversion efficiency of 5.08 % for a device with active area of larger than 0.3 cm^2^. Given that a simple planar structure was employed here and the optimization process was only performed on the active layer, we believe that further tuning of the developed process will allow for obtaining a higher PCE, using a commercially scalable, fast, and low-cost technique, i.e., spray coating.

## References

[1] Heo JH, Hyuk S, Noh JH, Mandal TN, Lim CS, Chang JA (2013). Efficient inorganic–organic hybrid heterojunction solar cells containing perovskite compound and polymeric hole conductors. Nature Photonics.

[2] Qinglong J, Xia S, Bing S, Xinjian F, Tao X (2014). Nickel-cathoded perovskite solar cells. J. Phys. Chem. C.

[3] Correa Baena JP, Steier L, Tress W, Saliba M, Neutzner S, Matsui T (2015). Highly efficient planar perovskite solar cells through band alignment engineering. Energy Environ. Sci..

[4] Chen T, Neutzner C, Colella S, Marras L, Srimath Kandad AR, Gandini M (2015). 17.6% stabilized efficiency in low-temperature processed planar perovskite solar cells. Energy Environ. Sci.

[5] Eperon GE, Burlakov VM, Docampo P, Goriely A, Snaith HJ (2014). Morphological control for high performance, solution- processed planar heterojunction perovskite solar cells. Adv. Funct. Mater..

[6] Schmidt MT, Larsen-Olsen TT, Carle JE, Angmo D, Krebs FC (2015) Upscaling of perovskite solar cells: fully ambient roll processing of flexible perovskite solar cells with printed back electrodes. Adv Eneregy Mater. doi:10.1002/aenm.201500569

[7] Frost JM, Butler KT, Brivio F, Hendon CH, Schilfgaarde MV, Walsh A (2014). Atomistic origins of high-performance in hybrid halide perovskite solar cells. Nano Lett..

[8] Zhu K, Miyasaka T, Kim JY, Mora-Sero M (2015). Trend of perovskite solar cells: dig deeper to build higher. J. Phys. Chem. Lett..

[9] Laban WA, Etgar A (2013). Depleted hole conductor-free lead halide iodide heterojunction solar cells. Energy Environ. Sci..

[10] Qin P, Tanaka S, Ito S, Tetreault N, Manabe K, Nishino H, Nazeeruddin MK, Gratzel M (2014) Inorganic hole conductor-based lead halide perovskite solar cells with 12.4 % conversion efficiency. Nat Commun 5:3834 10.1038/ncomms4834 24815001

[11] Kojima CR, Teshima K, Shirai Y (2009). Organomatal halide perovskite as visible-light synthesizer for photovoltaic cells. J Am Chem Soc.

[12] Aldibaja FK, Badia L, Mas-Marz E, Sanchez RS, Barea EM, Mora-Sero I (2015). Effect of different lead precursors on perovskite solar cell performance and stability. J. Mater. Chem. A..

[13] Pool VL, Gold-Parker A, McGehee MD, Toney MF (2015). Chlorine in PbCl_2_-derived hybrid-perovskite solar absorbers. Chem. Mater..

[14] Di Giacomo F, Razza S, Matteocci F, D Epifanio A, Licocci S, Brown MT (2014). High efficiency CH_3_NH_3_PbI_(3-x)_Cl_x_ perovskite solar cells with poly(3-hexylthiophene) hole transport layer. J. Power Sources.

[15] Dou L, Yang YM, You J, Hong Z, Chang WH, Li G et al (2014) Solution-processed hybrid perovskite photodetectors with high detectivity. Nat Commun 5:5404 10.1038/ncomms6404 25410021

[16] Lee MM, Teuscher J, Miyasaka T, Murakami TN, Snaith HJ (2012). Efficient hybrid solar cells based on meso-superstructured organometal halide perovskites. Science.

[17] Zhou H, Chen Q, Li G, Luo S, Song TB, Duan HS (2014). Interface engineering of highly efficient perovskite solar cells. Science..

[18] Liu M, Johnston MB, Snaith HJ (2013). Efficient planar heterojunction perovskite solar cells by vapour deposition. Nature..

[19] Pellegrino G, Colella S, Deretzis I, Condorelli GG, Smecca E, Gigli G (2015). Texture of MAPbI3 layers assisted by chloride on flat TiO2 substrates. The J. Phys. Chem. C..

[20] Eperon GE, Stranks S, Menelaou C, Johnston MB, Herz LM, Snaith HJ (2014). Formamidinium lead trihalide: a broadly tunable perovskite for efficient planar heterojunction solar cells. Energy Environ. Sci..

[21] Barrows AT, Pearson AJ, Kwak CK, Dunbar AD, Lidzey AR (2014). Efficient planar heterojunction mixed-halide perovskite solar cells deposited via spray deposition. Energy Environ. Sci..

[22] Wu Y, Islam A, Yang X, Qin C, Liu J, Zhang K (2014). Retarding the crystallization of PbI2 for highly reproducible planar-structured perovskite solar cells via sequential deposition. Energy Environ. Sci..

[23] Chen Q, Zhou H, Hong Z, Luo S, Duan HS, Wang HH (2014). Planar heterojunction perovskite solar cells via vapor-assisted solution process. J. Am. Chem. Soc..

[24] You J, Hong Z, Yang YM, Chen Q, Cai M, Song TB (2014). Low-temperature solution-processed perovskite solar cells with high efficiency and flexibility. ACS Nano..

[25] Jeng JY, Chiang YF, Lee MH, Peng SR, Guo TF, Chen P (2013). CH_3_NH_3_PbI_3_ perovkite/fullerene planar-heterojunction hybrid solar cells. Adv Mater.

[26] Chen CC, Bae SH, Chang WH, Hong Z, Li G, Chen Q, Zhou H, Yang Y (2015) Perovskite/polymer monolithic hybrid tandem solar cells utilizing a low-temperature, full solution process, Mat Horiz. doi:10.1039/c4mh00237g

[27] Burschk J, Pellet N, Moon SJ, Humphry-Baker R, Gao P, Nazeeruddin MK (2013). Sequential deposition as a route to high-performance perovskite-sensitized solar cells. Nature..

[28] Chen S, Lei L, Yang S, Liu Y, Wang ZS (2015). Characterization of perovskite obtained from two-step deposition on mesoporous titania. ACS Appl Mater Interfaces.

[29] Cohen BE, Gamliel S, Etgara L (2014). Parameters influencing the deposition of methylammonium lead halide iodide in hole conductor free perovskite-based solar cells. APL Mat..

[30] Bag M, Jiang Z, Renna LA, Jeong SP, Rotello VM, Venkataraman D (2016). Rapid combinatorial screening of inkjet-printed alkyl-ammonium cations in perovskite solar cells. Mater. Lett..

[31] Eslamian M (2014). Spray-on thin film PV solar cells: advances, potentials and challenges. Coatings..

[32] Eslamian M, Zabihi F (2015). Ultrasonic substrate vibration-assisted drop casting (SVADC) for the fabrication of solar cell arrays and thin film devices. Nanoscale Res Lett.

[33] Zabihi F, Xie Y, Gao S, Eslamian M (2015). Morphology, conductivity and wetting characteristics of PEDOT:PSS thin films deposited by spin and spray coating. App. Surf. Sci..

[34] Xie Y, Gao S, Eslamian M (2015). Fundamental study on the effect of spray parameters on characteristics of P3HT:PCBM active layers made by spray coating. Coatings.

[35] Zabihi F, Eslamian M (2015). Substrate vibration-assisted spray coating (SVASC): significant improvement in nano-structure, uniformity, and conductivity of PEDOT:PSS thin films for organic solar cells. J. Coatings Technol. Res..

[36] Habibi M, Eslamian M, Soltani-Kordshuli F, Zabihi F (2015) Controlled wetting/dewetting through substrate vibration-assisted spray coating (SVASC). J Coatings Technol Res. doi:10.1007/s11998-015-9748-2

[37] Wang Q, Eslamian M (2016) Improving uniformity and nanostructure of solution-processed thin films using ultrasonic dubstrate vibration post treatment (SVPT). Ultrasonics 67:55-64 10.1016/j.ultras.2015.12.012 26775261

[38] Leguy AMA, Hu Y, Campoy-Quiles M, Isabel Alonso M, Weber OJ, Azarhoosh P (2014). Reversible hydration of CH_3_NH_3_PbI_3_ in films, single crystals, and solar cells. Chem. Mater.

[39] Yu H, Wang F, Xie F, Li W, Chen J, Zhao N (2014). The role of chlorine in the formation process of “CH_3_NH_3_PbI_3-x_ Cl_x_” perovskite. Adv. Funct. Mater..

[40] Jeon NJ, Noh JH, Kim YC, Yang WS, Ryu S, Seok SI (2014). Solvent engineering for high-performance inorganic-organic hybrid perovskite solar cells. Nat. Mater.

[41] Mei A, Li X, Liu L, Ku Z, Liu T, Rong Y (2014). A hole-conductor*–*free, fully printable mesoscopic perovskite solar cell with high stability. Science.

[42] Habibi M, Zabihi F, Ahmadian-Yazdi MR, Eslamian M (2016) Progress in emerging solution-processed thin film solar cells – Part II: Perovskite Solar Cells. Renewable & Sustainable Energy Reviews, Revision under review.

[43] Paek S, Cho N, Choi H, Jeong H, Lim JS, Hwang JY (2014). Improved external quantum efficiency from solution-processed (CH3NH3)PbI3 Perovskite/PC71BM planar heterojunction for high efficiency hybrid solar cells. J. Phys. Chem. C..

[44] Zhao Y, Zhu K (2014). Solution chemistry engineering toward high-efficiency perovskite solar cells. J. Phys. Chem. Lett..

[45] Wei H, Shi J, Xin X, Xiao J, Luo J, Dong J (2015). Enhanced charge collection with ultrathin AlOx electron blocking layer for hole-transporting material-free perovskite solar cell. Phys. Chem. Chem. Phys..

[46] Zhang J, Pauporte T (2015). Effects of oxide contact layer on the preparation and properties of CH3NH3PbI3 for perovskite solar cell application. J. Phys. Chem. C.

[47] Preda N, Mihut L, Baibarac M, Baltog I, Lefrant S (2006). A distinctive signature in the Raman and photoluminescence spectra of intercalated PbI_2_. Condens Matter.

[48] Chen LC, Chen JC, Chen CC, Wu CG (2015). Fabrication and properties of high-efficiency perovskite/PCBM organic solar cells. Nanoscale Res Lett.

